# Prognostic factors for overall survival after surgical resection in patients with thymic epithelial tumors: A systematic review and meta-analysis

**DOI:** 10.1097/MD.0000000000030867

**Published:** 2022-09-30

**Authors:** Jiaduo Li, Yaling Liu, Xiaohe Zhang, Xuguang Zheng, Guoyan Qi

**Affiliations:** a People’s Hospital of Shijiazhuang affiliated to Hebei Medical University - Center of Treatment of Myasthenia Gravis, Shijiazhuang, Hebei, China.

**Keywords:** overall survival, prognostic factors, surgery, thymic epithelial tumors

## Abstract

Thymic epithelial tumors (TETs) originate in the thymic epithelial cell, including thymoma and thymic carcinoma. Surgical resection is the first choice for most patients. However, some studies have shown that the factors affecting the prognosis of these patients are not consistent. To evaluate prognostic factors in patients with surgically resected thymic epithelial tumors, we performed a meta-analysis.

**Methods:**

We searched the Chinese biomedical literature database, Pubmed, Embase, Cochrane Library and other electronic databases. Studies including postoperative overall survival (OS) and predictors of TETs were included. We made a comprehensive analysis the hazard ratios (HRs) through a single proportional combination. HRs were combined using single proportion combinations.

**Results:**

The meta-analysis included 11,695 patients from 26 studies. The pooled OS was 84% at 5 years and 73% at 10 years after TETs operation. The age as continuous-year (HR 1.04, 95% confidence interval (CI) 1.02–1.04), incomplete resection (HR 4.41, 95% CI 3.32–5.85), WHO histologic classification (B2/B3 vs A/AB/B1 HR 2.76, 95% CI 1.25–6.21), Masaoka Stage (stage III/IV vs I/II HR 2.74, 95% CI 2.12–3.55,) were the poor prognostic factors.

**Conclusions:**

For patients with TETs after surgical resection, advanced age, incomplete resection, WHO classification B2/B3, and higher Masaoka stage are risk factors for poor prognosis.

## 1. Introduction

Thymic epithelial tumors (TETs) is a relatively rare solid tumor of the chest originating from the thymus epithelial cells. TETs include thymoma and thymic carcinoma.^[1]^ The total incidence of TETs in different countries varies from 0.13 to 0.17 per 100-thousand person-years.^[2–4]^ TETs most commonly originate in the anterior mediastinum in adults.^[5]^ The 5-year survival rate of thymoma patients is about 78%.^[6]^ Complete surgical resection is the primary method for the treatment of TETs. The surgery goal is complete removal of the lesion, total thymectomy, and ensuring complete excision of other tumors from adjacent and non-adjacent tissues.^[6]^

Owing to small sample sizes, single-center designs, and heterogeneous population, most studies that aimed to determine the prognostic factors for TETs have reported different results. In order to evaluate the 5-and 10-year overall survival of patients with thymoma, we performed this meta-analysis. At the same time, we summarized the potential prognostic factors of TETs after surgical resection to identify important prognostic factors.

## 2. Materials

We followed the Preferred Reporting Items for Systematic Reviews and Meta-analyses (PRISMA) guidelines when we performed a meta-analysis.^[7]^ The study protocol has been registered at PROSPERO, number CRD42021235876. The ethical approval was not necessary and waived.

### 2.1. Search method

The past data has already been searched in PubMed, Embase, Cochrane Library, and the Chinese biomedical literature database from their establishment until December 10, 2020 to identify potential studies. The search process involved using the following terms or keywords with different combinations of “thymic epithelial tumor,” “surgery,” and “prognosis.”

The detailed search strategy of PubMed is listed here: ((prognos*[Title/Abstract]) AND (((((((Thymic epithelial tumor*[Title/Abstract]) OR (Thymom*[Title/Abstract])) OR (Thymic tumo*[Title/Abstract])) OR (Thymic carcinoma*[Title/Abstract])) OR (Thymus Neoplasms[MeSH Terms])) OR (Thymic epithelial tumor [Supplementary Concept])) OR (Thymoma[MeSH Terms]))) AND (((((((surgical operations, operative[MeSH Terms]) OR (Thymectomy[MeSH Terms])) OR (Thymectom*[Title/Abstract])) OR (surgical[Title/Abstract])) OR (surger*[Title/Abstract])) OR (operate[Title/Abstract])) OR (operation[Title/Abstract])). We also reviewed the retrieved articles to determine the relevant reports. Two authors independently assessed the eligibility of the retrieved items, and they settled disagreements by discussion or, if necessary, by consulting a third author. We decided that the remaining articles, including full text and references, were relevant and we reviewed them.

### 2.2. Study eligibility assessment

Relevant literature was critically reviewed. Eligible studies were included in the review. The authors settled their differences through mutual discussion and consensus. The inclusion criteria were as follows:

1. Studies evaluating prognostic factors after surgical resection.
2. Postoperative histopathological type was thymoma or thymic carcinoma.
3. 5- and 10-year OS and prognostic factors after surgical resection were reported.

The time from surgery to last follow-up or all-cause death is what we consider as the OS.

The exclusion criteria were as follows:

1. Reviews, letters, laboratory studies, and animal experiments.
2. Articles published in languages other than English or Chinese.

### 2.3. Quality assessment

Two authors (JL and YL) independently evaluated the quality of individual cohort studies using the Newcastle-Ottawa Scale (NOS). Each study was evaluated on a scale of 1 to 9, based on three subscales: the quality of selection, comparability, and patients’ outcome.^[8]^ If the quality score of a study was ≥8, it was defined as a high-quality study. The authors resolved any inconsistencies by jointly reevaluating the original article.

### 2.4. Data extraction

Two authors (JL and YL) independently extracted data from selected studies and ensured that they conformed to predefined standardized formats. Each step of the disagreements were resolved by consulting a third author or by mutual discussion until consensus. The relevant information was carefully extracted from all eligible articles.

The first step was to record basic information such as the first author, study period, study type, and number of patients. Thereafter, we took out the survival data, including the patient population, median duration of follow-up, average age at surgical resection, median survival time after surgical resection, 5- and 10-year overall survival (OS), and prognostic factors. We took the hazard ratios (HRs) and the 95% CI directly from some of the articles.

Data of univariate Cox hazard regression analysis were selected first. Data of multivariate Cox risk regression analysis were collected if the univariate data were not available. Tierney suggested a methodology for calculating HRs and 95% CIs based on the Kaplan–Meier curve, where HRs and 95% CIs were not reported.^[9]^

### 2.5. Analysis of data and statistics

From all cohorts, we retrieved prognostic indicators connected to the outcomes. If the stated *P* value was < .05 or if the 95 percent CI for an HR did not overlap by 1, the prognostic factor was considered significant. The statistical techniques used in the article or the choice of covariables used in a single multivariable model were dissimilar. Therefore, data interpretation from several multivariable models can be deceiving. This study only describes the prognostic factors evaluated by univariate analysis in at least two cohorts.

The 5- and 10-year OS rates of individuals were normally distributed after logit transformation. We used the DerSimonian and Laird method to calculate the pooled 5- and 10-year OS rates with 95% Cis.^[10]^ Actuarial methods were used to estimate the 5- and 10-year OS rates and the 95% CIs not reported in individual literature based on the Kaplan–Meier curve data. Using HR as a statistic, the prognostic factors for OS were analyzed by meta-analysis. According to the heterogeneity between studies, a fixed- or random-effect model was adopted. Statistical heterogeneity was assessed using the I^2^ statistic.

Use of random-effect models was preferred when the heterogeneity statistic was more than 50%. Otherwise, fixed-effect models were preferred. Meta-regression methods were used to investigate the sources of heterogeneity.

The degree of adjustment for confounding factors, including NOS score, published year, sample size, and median/mean age, was assessed. Egger’s test and funnel symmetry evaluated the potential publication bias, if the *P* value is greater than 0.05, we can infer there is no publication bias.^[11]^ In the R software (R Foundation, Vienna, Austria) and in Stata 13.0 (StataCorp, College Station, TX) we used the meta-package for statistical analysis.^[12,13]^ A *P* value of .05 was used to determine statistical significance.

## 3. Results

**Figure 1.**
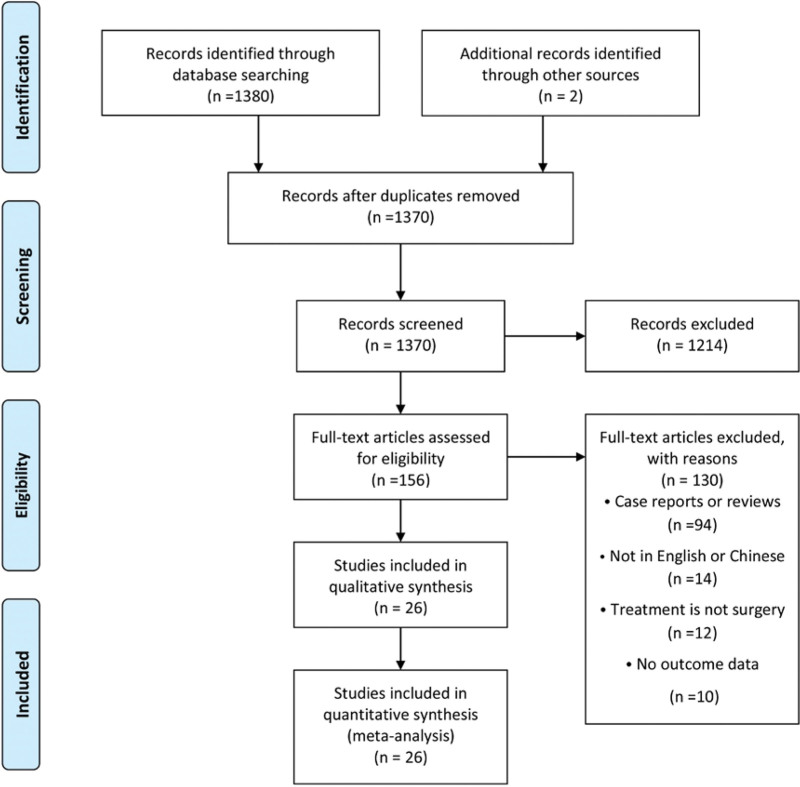
PRISMA flow diagram of the literature search process. PRISMA = preferred reporting items for systematic reviews.

After a preliminary search, we identified 1380 potentially related studies, including 805 in PubMed, 265 in Embase, 301 in the Chinese biomedical literature database, 7 in the Cochrane Library, and 2 via reference list review. Ten studies were excluded because of repetition, and additional 1214 studies were excluded after careful screening of titles and abstracts. The remaining 156 studies underwent full text review. Finally, 26 retrospective studies^[14–39]^ met all the inclusion criteria in the meta-analysis, with an average of 4498 patients in each study of the total 11695 patients (Fig. 1, Table 1).

**Table 1:** Characteristics of included studies.

Study	Area	Study duration	Study design	NOS	N	n	Follow-up (mo)	Age (yr)	Median Survival (months)	5-yr survival (%)	10-yr survival (%)	Excision type	Preoperative treatment	Postoperative treatment	Lymph node dissection	Mortality rates (%)	Death from TETs
Rieker et al, 2002	Germany	1967–1998	ROS	7	218	218	NR	50	NR	78.00	73	①②③	CT(3)	CT(30)	NR	32.1	37/70
													RT(2)	RT(68)			
														CT + RT(16)			
Kim et al, 2005	Korea	1992–2002	ROS	7	108	108	40.5	46.5	NR	80.20	71.1	①③④	NR	CT(1)	NR	18.5	14/20
														RT(30)			
														CT + RT(16)			
Chen et al, 2009	China	1997–2007	ROS	7	137	137	NR	35.1	NR	71.40	50.1	①②③	NR	NR	NR	32.8	NR
Margaritora et al, 2010	Italy	1972–2007	ROS	7	317	317	144.7	49	NR	89.90	84.1	①②③	NR	ALL(14)	NR	20.5	15/65
Sakamoto et al, 2012	Japan	1976–2009	ROS	7	162	162	NR	53	NR	94.70	85.7	①②③	CT(1)	CT(3)	NR	15.4	7/25
													RT(3)	RT(16)			
														CT + RT(1)			
Ruffini et al, 2014	European	1990–2010	ROS	6	2030	2030	48	56	NR	85.00	73	①②③	CT(170)	CT(44)	NR	15.9	NR
													RT(12)	RT(566)			
													CT + RT(67)	CT + RT(243)			
Guerrera et al, 2015	Italy	1990–2011	ROS	7	750	750	90	55	NR	91.00	77	①②③	ALL(105)	ALL(438)	NR	18.8	NR
Moon et al, 2015	Korea	1994–2010	ROS	7	437	437	NR	51	57	89.20	84.7	①②③④	NR	CT(34)	NR	15.3	56/67
														RT(191)			
														CT + RT(70)			
Lee et al, 2016	Korea	1994–2004	ROS	7	479	479	53	52	55	90.10	79.1	①②③	CT(44)	CT(204)	187	13.9	NR
													RT(2)	RT(12)			
														CT + RT(3)			
Nakajima et al, 2016	Japan	1991–2010	ROS	7	2334	2334	67.3	56.7	NR	92.00	85	NR	NR	NR	NR	6.2	43/145
Wang et al, 2016	China	1992–2012	ROS	8	1850	1850	NR	51.3	NR	89.10	81.4	①②③④⑤	NR	CT(353)	NR	NR	NR
														RT(803)			
Zhao et al, 2016	China	2001–2011	ROS	7	544	544	58	51.7	140.7	92.80	90.5	NR	NR	CT(14)	NR	NR	NR
														RT(240)			
														CT + RT(47)			
Tian et al, 2019	Japan	1976–2015	ROS	7	194	194	115	53.8	NR	92.70	87.5	NR	ALL(9)	ALL(79)	41	NR	NR
Alothaimeen et al, 2020	Saudi Arabia	1976–2014	ROS	7	56	56	65	39	NR	88.60	74.3	①②③	NR	NR	NR	14.2	8/8
Filosso et al, 2014	Italy	2000–2011	ROS	6	537	537	70	54	NR	88.00	75	①②③	ALL(53)	ALL(275)	NR	17.1	14/92
Gripp et al, 1998	Germany	1954–1991	ROS	7	70	70	85	46.5	183	71.00	58	①②③	NR	RT(22)	NR	50	25/35
														CT + RT(3)			
Chen et al, 2002	China	1969–1996	ROS	7	195	195	180	47	NR	79.00	69.4	NR	NR	CT(8)	NR	3.1	NR
														RT(55)			
Okuma et al, 2014	Japan	1976–2012	ROS	7	187	187	43.9	NR	NR	65.90	45.3	NR	NR	CT(44)	NR	NR	NR
														RT(22)			
Wilkins et al, 1999	America	1957–1997	ROS	8	136	136	NR	57	144	71.00	56	①②③	CT(1)	CT(9)	NR	44.1	19/60
													RT(3)	RT(44)			
Regnard et al, 1996	France	1955–1993	ROS	7	307	307	66	49	NR	82.10	67	③④⑤	NR	RT(139)	NR	29.9	32/92
Chalabreysse et al, 2002	France	1972–2001	ROS	7	90	90	NR	52	NR	74.20	NR	①②③	NR	CT(3)	NR	NR	NR
														RT(11)			
														CT + RT(26)			
Nakagawa et al, 2003	Japan	1962–2000	ROS	7	130	130	NR	54	NR	92.00	91	①③④	CT(4)	CT(1)	NR	25.3	11/33
														RT(6)			
Rea et al, 2004	Italy	1970–2001	ROS	7	132	132	92	50	NR	72.00	61	①②③	CT(32)	CT(24)	NR	38.6	NR
														RT(62)			
Jiao et al, 2008	China	1980–2005	ROS	7	108	108	62	50	NR	72.00	63	③④⑤	NR	NR	NR	24.1	20/26
Shen et al, 2013	China	2001–2006	ROS	7	115	115	72	64	NR	50.00	30	①②③	NR	CT(4)	NR	25.2	NR
														RT(44)			
														CT + RT(17)			
Moser et al, 2014	Austria	2001–2010	ROS	6	72	72	42.7	58.2	NR	87.00	64	①②③	NR	CT(20)	NR	NR	NR
														RT(33)			

①Complete thymectomy (thymectomy with resection of the surrounding fatty tissue).

②Complete extended thymectomy (thymectomy plus resection of neighboring structures, e.g., phrenic nerve, lymph nodes, parts of the pleura/ lung/pericardium/chest wall as well as arteries, veins and distant metastases).

③Incomplete resection (R1/R2 resection, explorative thoracotomy with biopsy, tumor debulking surgery, biopsy alone).

④Thymomectomy (the resection of thymoma leaving residual thymic tissue).

⑤Complete extended thymomectomy (thymomectomy plus resection of neighboring structures, e.g., phrenic nerve, lymph nodes, parts of the pleura/ lung/pericardium/chest wall as well as arteries, veins and distant metastases).

ALL = chemotherapy, radiotherapy and chemoradiotherapy, CT = chemotherapy, RT = radiotherapy, TETs = thymic epithelial tumors.

### 3.1. Study characteristics

Table 1 shows the detailed description of each characteristic of the patients identified from eligible clinical studies. All 26 studies had a retrospective design; among them, 20 were single-centric and 6 were multi-centric. Twenty-three studies have been published since 2001, and three studies were published before 2001.

Eighteen studies^[14,17–24,26,28–31,33,35,38,39]^ listed their median follow-up period (40.5–180 mo).

The range of age in patients undergoing surgical resection in the studies was from 35.1 to 64 years. The cohort size of 4 studies^[14,15,19,26]^ comprised <100 patients.

### 3.2. Evaluation of the included studies’ quality

Table 2 lists the quality evaluation of each study. The NOS was used to evaluate the included cohort study; it included eight items divided into three aspects (selection, comparability, results). In most studies, scores of 6 or 7 were common.

**Table 2:** Assessment of the quality of cohort studies using the Newcastle-Ottawa Scale (NOS).

Study	Selection													Comparability		Outcome										score
	a				b			c				d		e		f				g		h				
Rieker et al, 2002		*			*			*						*			*			*		*				7
Kim et al, 2005	*				*			*						*			*			*		*				7
Chen et al, 2009	*				*			*						*			*			*		*				7
Margaritora et al, 2010	*				*			*						*			*			*		*				7
Sakamoto et al, 2012		*			*			*						*			*			*		*				7
Ruffini et al, 2014	*		`		*			*						*			*			*						6
Guerrera et al, 2015		*			*			*						*			*			*		*				7
Moon et al, 2015	*				*			*						*			*			*		*				7
Lee et al, 2016	*				*			*						*			*			*		*				7
Nakajima et al, 2016	*				*			*						*			*			*		*				7
Wang et al, 2016	*				*			*						*	*		*			*		*				8
Zhao et al, 2016		*			*			*						*			*			*		*				7
Tian et al, 2019		*			*			*						*			*			*		*				7
Alothaimeen et al, 2020		*			*			*						*			*			*		*				7
Filosso et al, 2014		*			*			*						*			*					*				6
Gripp et al, 1998	*				*			*						*			*			*		*				7
Chen et al, 2002	*				*			*						*			*			*			*			7
Okuma et al, 2014	*				*			*						*			*			*		*				7
Wilkins et al, 1999	*				*			*						*	*		*			*			*			8
Regnard et al, 1996	*				*			*						*			*			*			*			7
Chalabreysse et al, 2002	*				*			*						*	*		*					*				7
Nakagawa et al, 2003	*				*			*						*			*			*		*				7
Rea et al, 2004	*				*			*						*			*			*		*				7
Jiao et al, 2008	*				*			*						*			*			*		*				7
Shen et al, 2013	*				*			*						*			*			*		*				7
Moser et al, 2014	*				*			*						*			*					*				6

a, The exposed cohort’s representativeness: high or somewhat representative (one star) of the exposed cohort; no description (no star).

b, Patients drawn from the same population as the exposed cohort (one star); patients drawn from a different source or no description for the nonexposed cohort (no star).

c, Exposure determination: data gathered from a secure record or structured interview (one star); no description (no star).

d, Yes (one star), no (no star): evidence indicating the desired outcome was not present at the start of the study.

e, Cohort comparability based on study design or analysis (all factors were included, two stars; some of them were included, one star).

f, Independent blind evaluation or record linkage (one star); self-report or no description (no star).

g, Follow-up for a long enough time to see results: yes (one star); no (no star).

h, Adequacy of cohort follow-up: complete follow up (one star); follow-up rate < 80% and no description of those lost (no star); no statement (no star).

### 3.3. OS and verall prognostic factors

**Figure 2.**
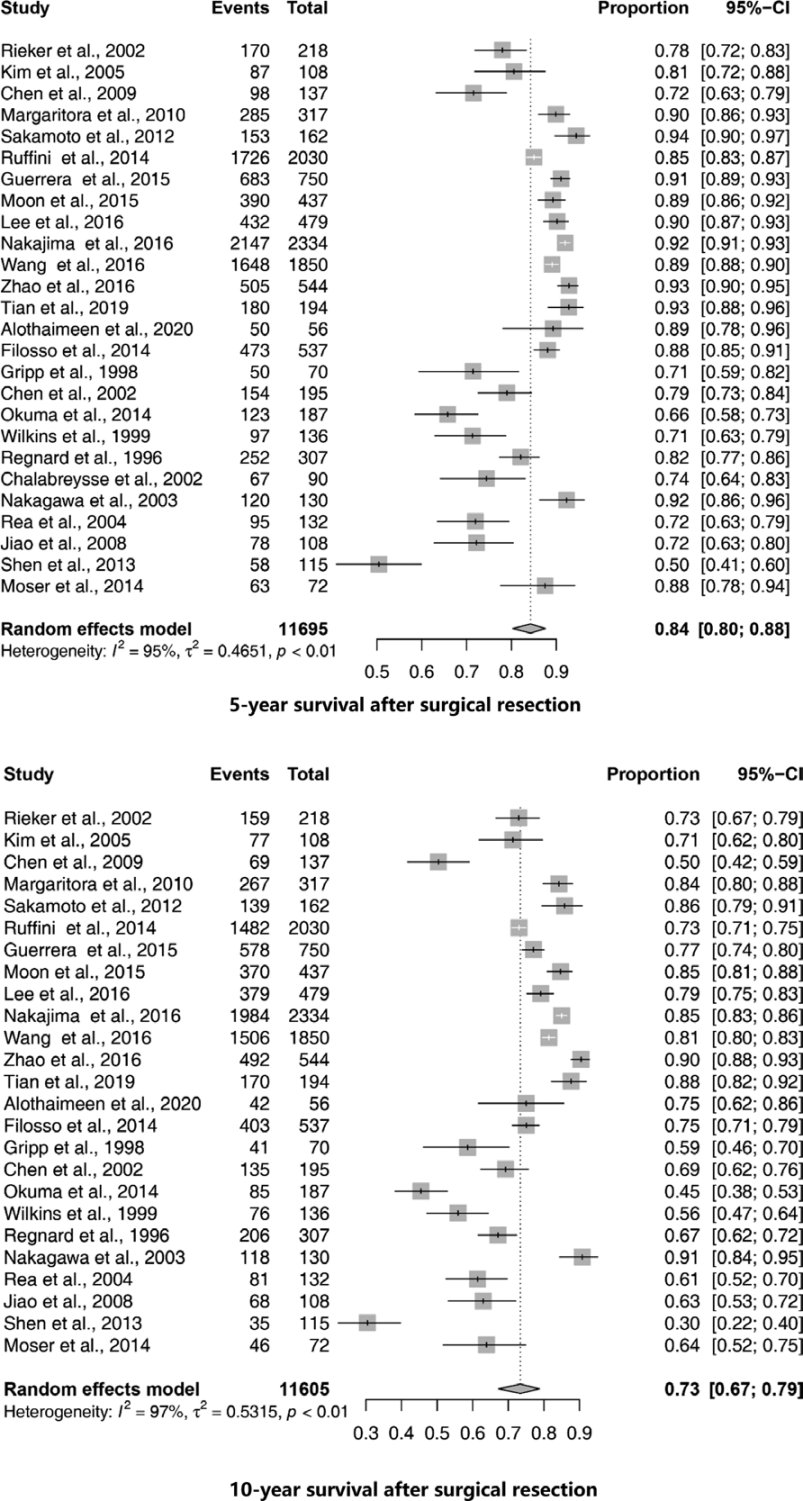
Forest plots showing 5, 10-year survival in each study. Each square represents an individual survival, with the size of the square being proportional to the weight given to the study. The dotted and dashed vertical lines represent combined survival for the whole population.

The median survival time of all patients was 55 to 183 months (Table 1). When pooled together, the 5-year OS rates were 84% (95% CI, 80–88%) and the 10-year OS rates were 73% (95% CI, 67–79%), respectively (Fig. 2).

Nine prognostic factors were identified in at least two different studies (Table 3).

**Table 3:** Univariate analyses were reported in two or more cohorts for the following prognostic factors.

Prognostic.factors	Number/significant*
Age	
Age ≥50 vs <50 yr	2/0
Age 50-59 vs <50 yr	1/0
Age 60-69 vs <50 yr	1/1 (34)
Age >70 vs <50 yr	1/1 (34)
Age (continuous, per 5 yr increase)	1/1 (33)
Age (as continous—yr)	6/5 (16,18,23,25,35)
Age ≥45 vs <45 yr	1/0
Age ≥57 vs <57 yr	1/1 (37)
Age 45-59 vs <45 yr	1/1 (15)
Age ≥60 vs <45 yr	1/1 (15)
Age ≥65 vs <65 yr	1/0
Gender (Male vs Female)	22/3 (25,33,38)
Myasthenia gravis (yes vs no)	23/5 (25,32–34,37)
Type of resection	
Incomplete vs complete	19/15 (14–16,18,20–22,24,26,28,30,32,33,36,37)
R1 vs R0	2/1 (23)
R2 vs R0	2/1 (25)
R2 vs R1	1/0
Tumour size	
>8 vs ≤8 cm	1/1 (32)
Continuous, per 1 cm increase	4/2 (23,33)
≥5 cm vs <5 cm	1/0
≥7.3 cm vs <7.3 cm	1/0
Masaoka–Koga stage	
II vs I	8/3 (22,32,38)
III vs I	11/11 (17,22–25,29,30,32,33,36,38)
IV vs I	9/9 (22–25,30,32,33,36,38)
III/IV vs I II	4/4 (16,18,28,39)
III vs II	5/5 (21,24,25,29,31)
IV vs II	2/2 (24,25)
IV vs III	4/3 (17,21,25)
III vs I II	2/1 (20)
IV vs I II	2/1 (20)
Histology (WHO)	
C vs A/AB/B1/B2	2/2 (22,32)
B2/B3/C vs A/AB/B1	3/2 (16,17)
B2/B3 vs A/AB/B1	6/4 (21,25,33,39)
C vs A/AB/B1	3/2 (25,33)
C vs A/AB/B1/B2/B3	2/2 (14,15)
C vs B1	2/2 (30,32)
C vs B3	2/2 (21,32)
Adjuvant treatment	12/3 (23,25,35)
Surgical approach	3/0

* The number of studies in which the factor was measured/Number of studies in which significant association with poor outcome was reported (log-rank test, a < 0.05).

#### (1) Effect of sex, age, and presence of myasthenia gravis on OS

Sixteen studies^[14,17–20,22–25,28,32,33,35–38]^ assessed the impact of sex on OS, and only 3^[25,33,38]^ concluded that it significantly affected OS.

**Figure 3.**
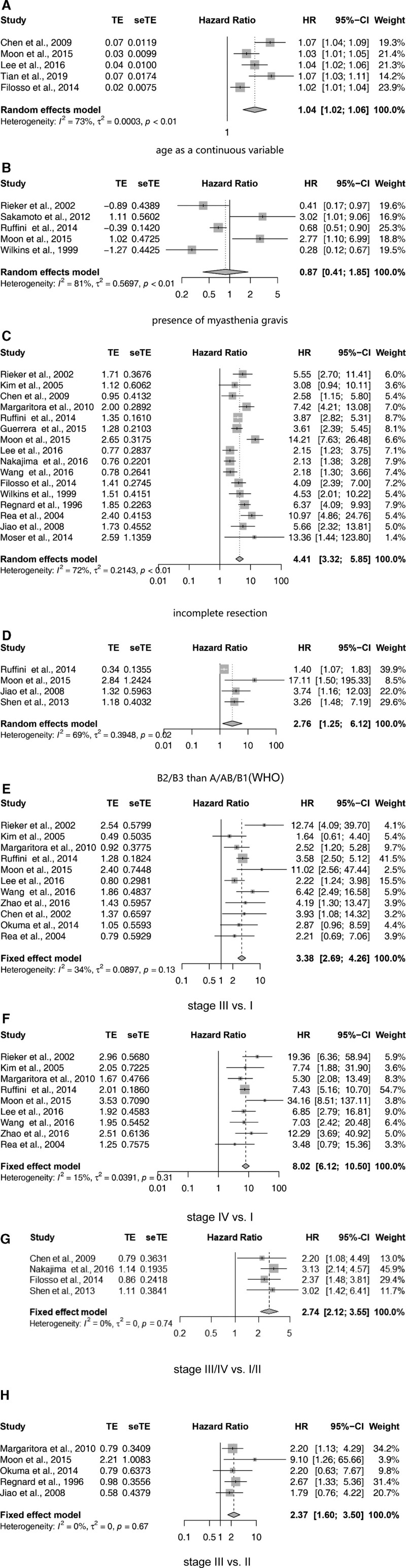
Overview of calculated hazard ratios (HR) for: (A) age as a continuous variable; (B) presence of myasthenia gravis; (C) incomplete resection; (D) B2/B3 than A/AB/B1; (E) stage III than stage I tumors; (F) stage IV than stage I tumors; (G) stage III/IV than stage I/II tumors; (H) stage III than stage II tumors.

Similarly, 9^[15,16,18,23,25,33–35,37]^ of 14 studies^[14–16,18,19,23–25,32–37]^ concluded that age had a significant impact on OS. A meta-analysis of 5 studies^[16,18,23,25,35]^ assessing age as a continuous variable showed that age was correlated with negative outcomes (HR, 1.04; 95% CI, 1.02–1.04; *P* < .001) (Fig. 3A).

Five^[25,32–34,37]^ of the 17 studies^[14,16,18–20,22–25,28,32–38]^ showed that the presence of myasthenia gravis was a prognostic factor for OS. Upon meta-analysis, we concluded that myasthenia gravis has no effect on OS (HR, 0.87; 95% CI, 0.41–1.85; *P* = .7) (Fig. 3B).

#### (2) Effect of adjuvant treatment, surgical approach, and resection status on OS

Three^[23,25,35]^ of the 12 studies^[14,17–19,23,25,30,31,35–38]^ reported that adjuvant therapy was a prognostic factor for OS. Tian et al’s results suggested that preoperative induction therapy was an independent prognostic factor for OS. The results of Lee et al suggested that preoperative chemotherapy was a predictor of recurrence after R0 resection. Moon et al believe that the history of adjuvant chemotherapy and simultaneous concurrent chemoradiation therapy were the factors for the poor prognosis of OS. All three studies showed that surgical approach was not a prognostic factor for OS. Only two studies^[23,35]^ reported lymph node dissection.

A total of 21^[14–16,18–26,28,30–38]^ studies assessed the impact of resection status on OS, and 16^[16,18,20–26,28,30–33,36,37]^ of these studies concluded that resection status significantly affected OS. We performed a meta-analysis on these 16 trials and discovered that inadequate resection may indicate a poor prognosis (HR, 4.41; 95% CI, 3.32–5.85; *P* < .001) (Fig. 3C).

#### (3) Prognostic variables for the tumor’s prognosis

Three^[23,32,33]^ of 7 studies^[23,28,32,33,35,36,38]^ suggested that tumor size might significantly influence OS. Seventeen^[14,16–20,22–25,28,32–36,38]^ studies assessed the impact of World Health Organization (WHO) histologic classification on OS. A meta-analysis of four of these research^[21,25,33,39]^ found that B2/B3 thymoma was associated with a worse prognosis than A/AB/B1 thymoma (HR, 2.76; 95% CI, 1.25–6.21; *P* = .01) (Fig. 3D).

A meta-analysis of four of these studies with survival data showed that C thymoma was related to poorer OS than others (HR, 4.97; 95% CI, 3.88–6.38; *P* = .25) (Fig. 3J).

A total of 21^[14,16–18,20–25,28–34,36–39]^ studies evaluated the Masaoka Stage, and 11^[17,22–25,29,30,32,33,36,38]^ of these studies reported that stage III disease might confer poorer OS than stage I tumors (HR, 3.38; 95% CI, 2.69–4.26; *P* < .001) (Fig. 3E).

Nine^[22–25,30,32,33,36,38]^ of these studies revealed that stage IV disease might confer poorer OS than stage I disease (HR, 8.02; 95% CI, 6.12–10.50; *P* < .001) (Fig. 3F), 4^[16,18,28,39]^ of these revealed that stage III/IV disease might confer poorer OS than stage I/II disease (HR, 2.74; 95% CI, 2.12–3.55; *P* < .001) (Fig. 3G), and 5^[21,24,25,29,31]^ of these studies revealed that stage III disease might confer poorer OS than stage II disease (HR, 2.37; 95% CI, 1.60–3.50; *P* < .001) (Fig. 3H).

We found that heterogeneity was present in the analysis of prognostic factors, including age, presence of myasthenia gravis, resection status, and WHO histologic classification. The effect sizes of the original studies were assessed after adjusting for study year, sample size, NOS score, and mean age. In any meta-analysis of these predictive factors, confounders were unable to explain the heterogeneities.

### 3.4. Examination of publication bias

Neither Begg’s nor Egger’s test found evidence of publication bias in either 5- or 10-year survival.

## 4. Discussion

Our study analyzed the results of similar studies to find survival rates of patients who had TETs. Few similar studies have been conducted. This study provides reliable information by evaluating more than 11000 patients with TETs who underwent surgery. Surgical resection has become a routine treatment for TETs. The pooled 5- and 10-year OS rates after TET resection were 84 percent and 73%, respectively, in this meta-analysis. Overall, this prognosis was good because most patients were eligible for surgery. These surgical candidates are a highly selective group with a high level of performance and a low risk of disease. Previous studies investigating the prognostic factors for survival in postoperative patients with TETs reported inconsistent results. Identifying the prognostic factors that can significantly improve the survival rate remains a problem in thoracic surgery.

Univariate analysis data were published in at least two studies, and our investigation collected all predictive factors from the 26 included cohorts. Meta-analysis showed that Masaoka stage, WHO histological type, age and resection were related to postoperative OS in patients with TETS. Incomplete resection implied a dismal prognosis as it likely reflects progressive disease with tumor dissemination. These patients usually have a poorer prognosis, which may be related to a greater tumor burden and a larger number of cancer cells left behind after surgery. Age was also related to an unfavorable survival rate. Of note, WHO histologic classification, especially B2/B3 thymoma, was a prognostic factor for OS in TET patients after surgical resection.

Moreover, we found that a higher Masaoka stage was an indicator of poor prognosis.

Unlike previous studies that addressed the role of myasthenia gravis as an adverse factor in survival,^[40,41]^ our results indicate that myasthenia gravis has no impact on survival. Based on our findings, we recommend that all cases be reviewed by the multidisciplinary oncology committee. It can provide multi-mode treatment for patients with Masaoka stage III and IV and thymoma who can not be completely resected. For patients with the above risk factors, we recommend a lifetime annual follow-up through CT. Due to the relatively small number of patients receiving thymic epithelial tumor treatment in each surgical center around the world, we recommend that studies and reports be based on consistent reporting standards, especially the description of some clinical data details.

Our research, however, has a number of flaws. Firstly, the results of meta-analysis may be influenced by the quality of individual study. It is necessary to identify and quantify these factors before arriving at a conclusion. Our quality assessment based on NOS, 2 of the 26 studies scored 8, 21 scored 7, and the other 3 scored 6, indicating that all the studies were of medium quality. Another downside was that the extrapolation of HR might be biased. We have calculated the missing statistics if the authors did not report them. If that information wasn’t available, The results would have been extrapolated using Kaplan–Meier curves. As a result, some subjective facts may have an impact on the outcome. Thirdly, the search filter only searches for articles that have been published, which might cause a publication bias. Another potential limitation of our review is that only English and Chinese language papers were screened for.

## 5. Conclusions

An older age, incomplete resection, WHO classification B2/B3, and higher stage are risk factors for predicting poor survival in TET patients after surgical resection. Future investigations need to include both of these aspects.

## Acknowledgments

We thank all of the participants recruited for this study.

## Authors contributions

Conceptualization: Yaling Liu.

Data curation: Yaling Liu.

Formal analysis: Yaling Liu.

Funding acquisition: Yaling Liu.

Investigation: Xiaohe Zhang.

Methodology: Xiaohe Zhang.

Project administration: Xiaohe Zhang.

Resources: Xiaohe Zhang, Xuguang Zheng.

Software: Xuguang Zheng.

Supervision: Xuguang Zheng.

Validation: Xuguang Zheng.

Visualization: Xuguang Zheng.

Writing – original draft: Jiaduo Li.

Writing – review & editing: Guoyan Qi.
